# Aortic dimensions on cardiovascular magnetic resonance imaging relate to pregnancy outcomes in women with coarctation of the aorta: a multicenter study

**DOI:** 10.1186/1532-429X-14-S1-O68

**Published:** 2012-02-01

**Authors:** Laura Jimenez Juan, Eric Krieger, Anne Marie Valente, Julia Ley-Zaporozhan, Hadas Moshonov, Bernd J Wintersperger, Candice Silversides, Sam Siu, Andrew M Crean, Sebastian Ley, Jack M Colman, Elsie T Nguyen, Narinder S Paul, Mathew Sermer, Rachel M Wald

**Affiliations:** 1 Medical Imaging, Toronto General Hospital, Toronto, ON, Canada; 2 Cardiology, Children’s Hospital Boston, Boston, MA, USA; 3 Cardiology, Toronto General Hospital, Toronto, ON, Canada; 4 Cardiology, Mount Sinai Hospital, Toronto, ON, Canada; 5 Cardiology, London Health Sciences Centre, London, ON, Canada; 6 Obstetrics and Gynecology, Mount Sinai Hospital, Toronto, ON, Canada

## Summary

To examine the association between aortic dimensions on cardiovascular magnetic resonance imaging (CMR) and risk of adverse events related to pregnancy in women with coarctation of the aorta (CoA).

## Background

Women with CoA are at increased risk of developing complications during pregnancy. The relationship between CMR measures of the aorta and pregnancy outcome is unknown.

## Methods

Consecutive women seen in several tertiary care institutions with CMR studies (including contrast enhanced-MR angiography) within 2 years of pregnancy were included. Those with aortic stents were excluded. 28 women with CoA (previously repaired n=24 and unrepaired n=4) who had 30 pregnancies were reviewed. Aortic diameters and cross-sectional areas were systematically measured at various predefined levels from the ascending aorta to the level of the diaphragm. Cardiovascular events (hypertension, sustained arrhythmia, heart failure, stroke, cardiac arrest, and/or need for an urgent cardiac procedure), obstetric complications (eclampsia, pre-term labour, post-partum hemorrhage) and fetal/neonatal events (still birth, prematurity, low birthweight, respiratory distress syndrome, intraventricular hemorrhage, death) were recorded.

## Results

**Figure 1:**
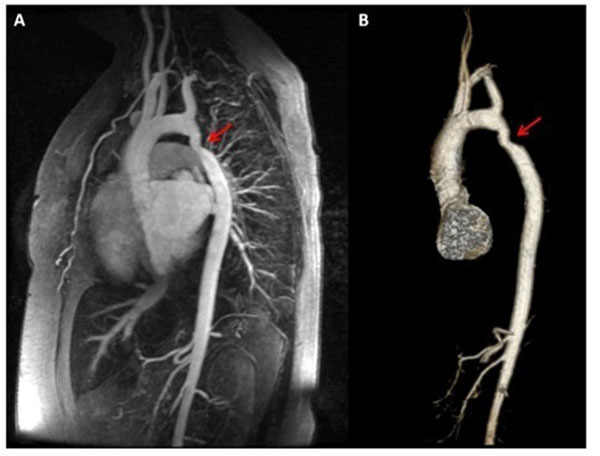
A 3D contrast enhanced-MR angiogram (panel A) and volume rendered image of the thoracic aorta (panel B) in a 42 year old female demonstrating unrepaired coarctation of the aorta with a minimum diameter of 11 cm. She presented with a stroke during pregnancy and subsequently went on toe have stillbirth.

Demographic characteristics and event rates are shown in table 1. A total of 8 cardiovascular events were seen in 6 pregnancies (6/30, 20%), obstetric complications in 1 pregnancy (1/30, 3%) and perinatal events in 4 pregnancies (4/30, 13%). Minimum arch dimensions were at the isthmus in 21/28 (75%) and at the transverse arch in 7/28 (25%). The minimum diameter and area of the aorta (absolute and indexed to body surface area) were smaller in women diagnosed with cardiovascular events during pregnancy compared with women without events (mean diameter 13.5±2.1 versus 15.5±2.0 mm, p=0.02; mean indexed diameter 7.7 ±2.0 versus 9.0±1.3 mm/m2, p=0.04; mean area 166.0±75.2 versus 207.9±48.5 mm2, p=0.03; mean indexed area 89.2±43.5 versus 117±27 mm/m2, p=0.04)(figure 1). The cardiovascular event rate in women with minimum arch diameter ≤13 mm was 60% (3/5) and in those with minimum arch diameter >13 mm was 12% (3/25)(p=0.02). Use of cardiovascular medications and age at initial intervention were not significantly different in those with and without cardiovascular events. Women with fetal/neonatal events had smaller arch diameters than those without events (12.5 [IQR 11.2-14.15] versus 15.0 mm [IQR 13.7-16.9], p=0.047).

**Table 1:** Demographic details and clinical events

Variable	
**Age**	**Mean ± SD**

Age at intervention (years) (n=28)	6.5 ±8.1
Age at pregnancy (years) (n=30)	29.2±64
Age at time of CMR (years) (n=31)	30.6±5.9

**Number of pregnancies (n=28)**	**n(%)**

1 pregnancy	15 (54%)
2 pregnancies	7 (25%)
3 or more pregnancies	6 (21%)

**Type of cardiac lesion (n=28)**	

CoA alone	2 (7.1%)
CoA with bicuspid aortic valve	17 (60.7%)
CoA with additional complex lesion(s)	9 (32.1%)

**Type of Intervention (n=28)**	

Subclavian flap repair	8 (29%)
End to end anastomosis	14 (50%)
Balloon dilatation	2 (7%)
Unrepaired	4 (14%)

**Clinical events (n=30)**	

**Cardiovascular events related to pregnancy**	

Sustained arrhythmia requiring medical therapy	2 (6%)
Stroke	2 (6%)
Hypertension	4 (13%)

**Obstetric events**	

Postpartum hemorrhage	1 (3%)

**Fetal/neonatal events**	

Still birth	1 (3%)
Small for gestational age	1 (3%)
IUGR	1 (3%)
Prematurity	1 (3%)

## Conclusions

This is the first study to relate pregnancy outcome with morphologic features of the aortic arch in women with CoA. Smaller arch dimensions appear to be associated with cardiovascular and fetal/neonatal complications related to pregnancy. Detailed assessment of the aorta may help identify those at high risk during pregnancy.

## Funding

NA.

